# Predicting all-cause risk of 30-day hospital readmission using artificial neural networks

**DOI:** 10.1371/journal.pone.0181173

**Published:** 2017-07-14

**Authors:** Mehdi Jamei, Aleksandr Nisnevich, Everett Wetchler, Sylvia Sudat, Eric Liu

**Affiliations:** 1 Bayes Impact, Technology 501(c)(3) Non-profit, San Francisco, California, United States of America; 2 Research, Development and Dissemination, Sutter Health, Walnut Creek, California, United States of America; University of Waterloo, CANADA

## Abstract

Avoidable hospital readmissions not only contribute to the high costs of healthcare in the US, but also have an impact on the quality of care for patients. Large scale adoption of Electronic Health Records (EHR) has created the opportunity to proactively identify patients with high risk of hospital readmission, and apply effective interventions to mitigate that risk. To that end, in the past, numerous machine-learning models have been employed to predict the risk of 30-day hospital readmission. However, the need for an accurate and real-time predictive model, suitable for hospital setting applications still exists. Here, using data from more than 300,000 hospital stays in California from Sutter Health’s EHR system, we built and tested an artificial neural network (NN) model based on Google’s TensorFlow library. Through comparison with other traditional and non-traditional models, we demonstrated that neural networks are great candidates to capture the complexity and interdependency of various data fields in EHRs. LACE, the current industry standard, showed a precision (PPV) of 0.20 in identifying high-risk patients in our database. In contrast, our NN model yielded a PPV of 0.24, which is a 20% improvement over LACE. Additionally, we discussed the predictive power of Social Determinants of Health (SDoH) data, and presented a simple cost analysis to assist hospitalists in implementing helpful and cost-effective post-discharge interventions.

## Introduction

Since the Affordable Care Act (ACA) was signed into law in 2010, hospital readmission rates have received increasing attention as both a metric for the quality of care and a savings opportunity for the American healthcare system [1]. Per American Hospital Association, the national readmission rate finally fell to 17.5% in 2013 after holding at approximately 19% for several years [2]. Hospital readmissions cost more than $17 billion annually [3]. According to the Medicare Payment Advisory Committee (MedPAC), 76% of hospital readmissions are potentially avoidable [4].

In response, ACA has required the Center for Medicare and Medicaid Services (CMS) to reduce payments to hospitals with excess readmissions [5]. These penalties should be put in the context of a larger shift in healthcare from the current fee-for-service payment model to a more patient-centered value-based payment model. Formation of Accountable Care Organizations (ACO) and CMS’ Quality Payment Program are examples of this trend that has created financial incentives for hospitals and care providers to address the readmission problem more systematically.

Before establishing targeted intervention programs, it is important to first identify those patients with a high risk of readmission. Fortunately, the widespread adoption of EHR systems has produced a vast amount of data that could help predict patients’ risk of future readmissions. Numerous attempts to build such predictive models have been made [6–12]. However, the majority of them suffer from at least one of the following shortcomings: (1) the model is not predictive enough compared to LACE [11], the industry-standard scoring model [13], (2) the model uses insurance claim data, which would not be available in a real-time clinical setting [6,7], (3) the model does not consider social determinants of health (SDoH) [13,8], which have proven to be predictive [14], (4) the model is limited to a particular medical condition, and thus, limited in scope [9,10].

To address these shortcomings, we built a model to predict all-cause 30-day readmission risk, and added block-level census data as proxies for social determinants of health. Additionally, instead of using insurance claims data, which could take up to a month to process, we built our model on the data available during the inpatient stay or at the time of discharge. Generally, using real-time EHR data allows models to be employed in hospital setting applications. Particularly, the authors are interested in applications of this predictive model in supporting data-driven post-discharge interventions to mitigate the risk of hospital readmission.

## Methods

### Ethics

This study was conducted using health record data (without patient names) taken from 20 hospitals across Sutter Health, a large nonprofit hospital network serving Northern California. The Institutional Review Board (IRB) of Sutter Health (SH IRB # 2015.084EXP RDD) approved the study.

### Data preparation

**Fig 1:**
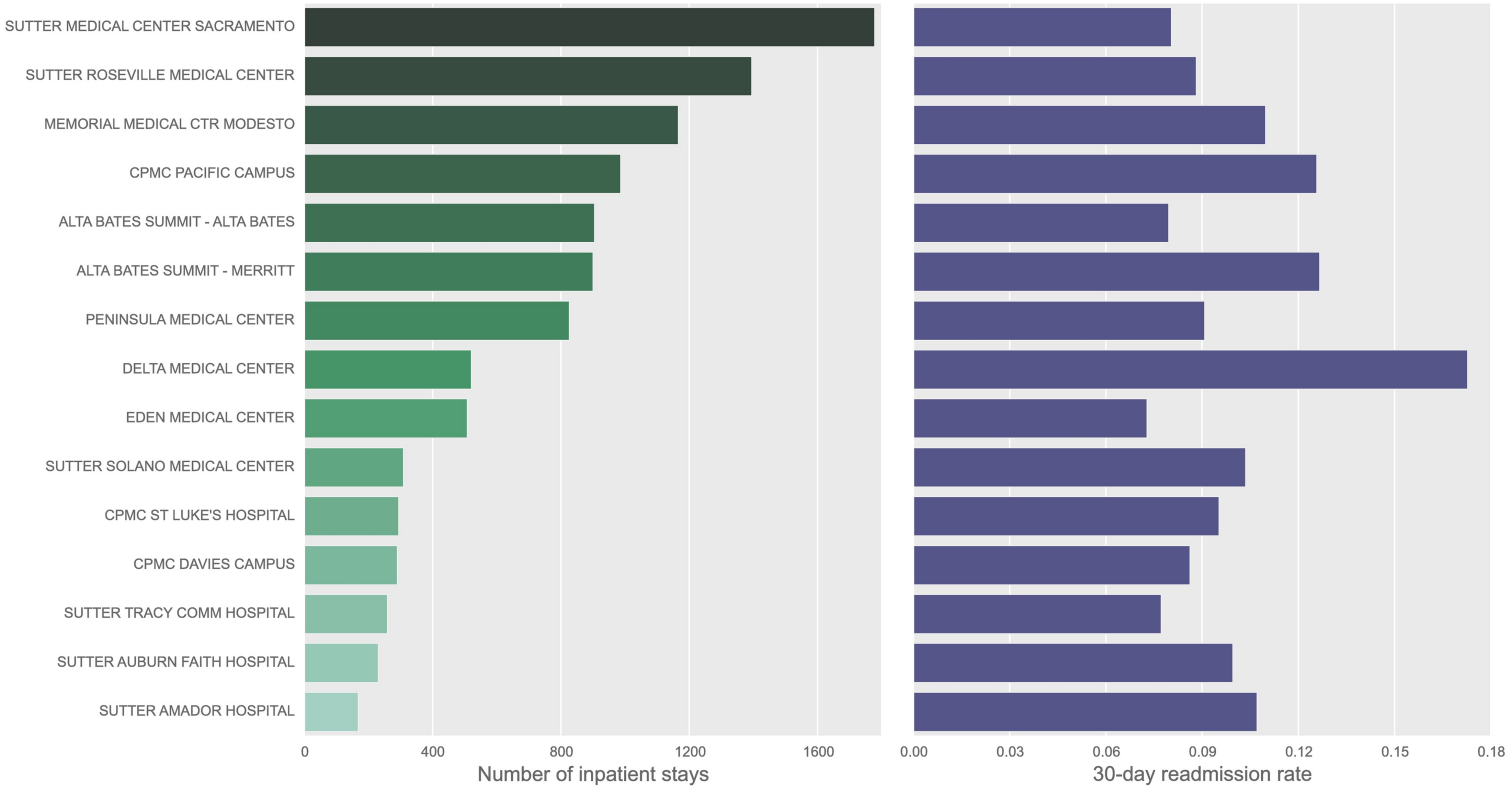
Total number of records for each hospital under study, and their respective readmission rates.

**Fig 2:**
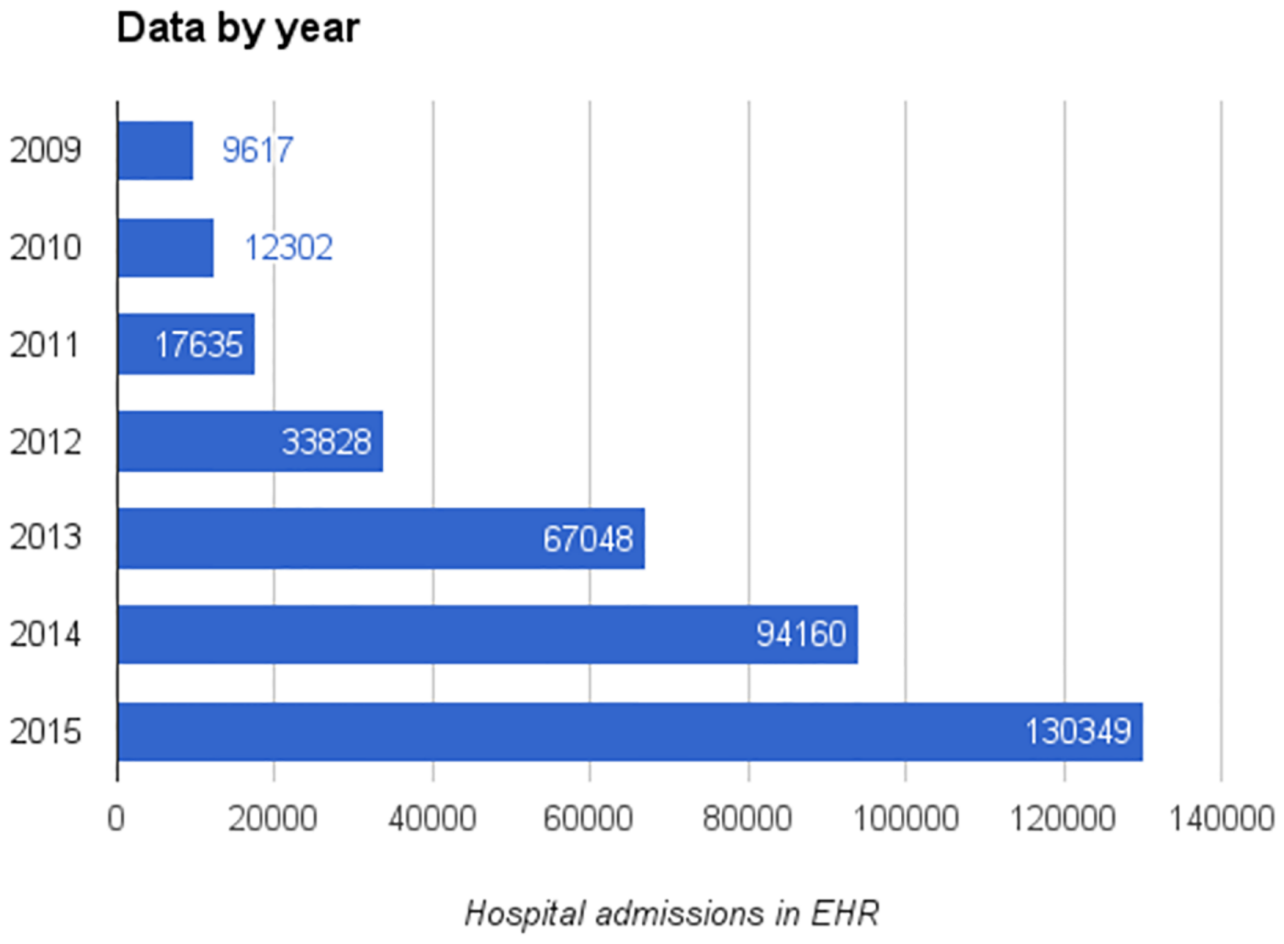
Data breakdown by hospital admission year.

Electronic health records corresponding to 323,813 inpatient stays were extracted from Sutter Health’s EPIC electronic record system. Table 1 shows a summary of the population under study. We had access to all Sutter EHR data, beginning in 2009 and going through the end of 2015. Since many hospitals only recently completed their EHR integration, some 80% of the data comes from 2013–2015 (Fig 1). To ensure data consistency, we limited our hospitals of study to those with over 3,000 inpatient records and excluded Skilled Nursing and other specialty facilities. Fig 2 shows the total number of records for each hospital, and their respective readmission rates.

**Table 1:** summary of the population under study.

Variable	All hospital visits (n = 335,815)	Visits resulting in 30-day readmission (n = 32,718)	Visits not resulting in 30-day readmission (n = 303,097)
**Admission source (%)**			
Home	93.0	91.8	93.1
Outpatient	0.1	0.1	0.1
Transfer	5.1	5.9	5.0
Other	1.8	2.1	1.7
**Admission type (%)**			
Elective	27.3	11.7	29.0
Emergency	43.2	58.2	41.6
Urgent	28.2	29.5	28.0
Other	1.4	0.6	1.4
**Age (%)**			
0–44	29.6	15.1	31.1
45–64	27.2	30.0	26.9
65–84	31.5	39.0	30.7
85+	11.7	15.9	11.3
**Alcohol users (%)**	28.3	25.3	28.6
**Charlson Comorbidity Index, median (IQR)**	1.0 (4.0)	4.0 (4.0)	1.0 (3.0)
**Discharge location (%)**			
Home or self care (routine)	70.4	56.2	71.9
Home under care of home health service organization	15.0	22.3	14.2
SNF	14.6	21.5	13.9
**Discharge time (%)**			
Morning (8:00 AM–12:59 PM)	25.9	19.1	26.7
Afternoon (1:00 PM–5:59 PM)	61.4	65.8	60.9
Evening (6:00 PM–7:59 AM)	12.6	15.1	12.4
**Drug users (%)**	6.5	8.3	6.3
**Female (%)**	61.9	54.6	62.7
**Hispanic of any race (%)**	17.5	13.8	17.9
**Insurance payer (%)**			
Commercial	46.1	36.6	47.1
Medicare	51.5	62.2	50.3
Self-pay	2.2	1.0	2.3
Other	0.2	0.2	0.2
**Interpreter needed (%)**	9.4	8.8	9.5
**LACE Score, median (IQR)**	6.0 (5.0)	10.0 (5.0)	6.0 (6.0)
**Length of stay in days, median (IQR)**	3.0 (3.0)	4.0 (5.0)	3.0 (3.)
**Marital status (%)**			
Single	27.2	28.6	27.0
Married/partner	48.2	39.6	49.1
Divorced/separated	8.9	11.4	8.6
Widowed	14.8	19.9	14.3
Other/unknown	0.9	0.4	0.9
**Previous emergency visits, mean (SD)**			
In the past 3 months	0.3 (1.0)	0.7 (1.6)	0.3 (0.9)
In the past 6 months	0.5 (1.5)	1.1 (2.4)	0.5 (1.4)
In the past 12 months	0.8 (2.4)	1.7 (3.9)	0.7 (2.1)
**Previous inpatient visits, mean (SD)**			
In the past 3 months	0.3 (0.7)	0.8 (1.3)	0.2 (0.6)
In the past 6 months	0.4 (1.1)	1.1 (2.0)	0.3 (0.9)
In the past 12 months	0.6 (1.5)	1.6 (3.0)	0.5 (1.2)
**Race (%)**			
White	61.9	61.9	61.9
Black	11.2	16.3	10.7
Other	25.9	21.2	26.4
**Tabak Mortality Score, median (IQR)**	25.5 (15.6)	32.0 (15.8)	24.7 (14.9)
**Tobacco users (%)**	12.4	15.2	12.1

We studied all inpatient visits to all Sutter hospitals. Hospital transfers and elective admissions were excluded. With this method, a 30-day boolean readmission label was created for each hospital admission.

In the current version of their EHR system, Sutter Health captures a few SDoH data fields, such as history of alcohol and tobacco use. We supplemented those data with block-level 2010 census data [15] by matching patients’ addresses. The Google Geocoding API was used to determine the coordinates of each patient’s home address, and a spatial join was performed with the open-source QGIS platform [16] to find respective census tract and block IDs.

The data was transferred from Sutter to a HIPAA-compliant cloud service, where it was stored in a PostgreSQL database. An open-source framework [17], written in Python, was built to systematically extract features from the dataset. In total, 335,815 patient records with 1667 distinct features, comprising 15 feature sets, were extracted from the database, as summarized in Table 2.

**Table 2:** Summary of extracted feature categories, and two sample features per category.

Category	Count	Sample features
**Encounter Reason**	604	abscess, kidney_stone
**Hospital Problems**	287	hcup_category_cystic_fibro, hospital_problems_count
**Procedures**	232	px_blood_transf, px_c_section
**Medications**	202	inp_num_unique _meds, outp_med_antidotes
**Provider**	119	specialty_orthopedic_surgery, specialty_hospitalist_medical
**Discharge**	46	length_of_stay, disch_location_home_no_service
**Socioeconomic**	44	pct_married, median_household_income
**Admission**	39	admission_source_transfer, admission_type_elective
**Lab Results**	26	num_abnormal_results, tabak_very_low_albumin
**Comorbidities**	19	charlson_index, comor_chf
**Basic Demographics**	16	age, if_female
**Health History**	11	alcohol_no, tobacco_quit
**Utilization**	10	pre_12_month_inpatient, pre_6_month_inpatient
**Vitals**	8	bmi, pulse
**Payer**	4	insurance_type_medicare, insurance_type_self-pay

Each type of feature (age, length of stay, etc) was independently studied using Jupyter Notebook, an interactive Python tool for data exploration and analysis. Using the pandas [18] library, we explored the quality and completeness of the data for each feature, identified quirks, and came to a holistic understanding of the feature, before using it in our models. Each feature-study notebook provided a readable document mixing code and results, allowing the research team to share findings with one another in a clear and technically reproducible way.

### Model training and evaluation

Initially, we experimented with several classic and modern classifiers, including logistic regression, random forests [19], and neural networks. In each case, a 5-fold cross validation, with 20% of the data kept hidden from the model, was performed. We found that the neural network models heavily outperformed other models in performance and recall, with the neural network model being about 10 times faster to train than the random forest model, the second best performing model. Therefore, we focused on optimizing the neural network model.

**Fig 3:**
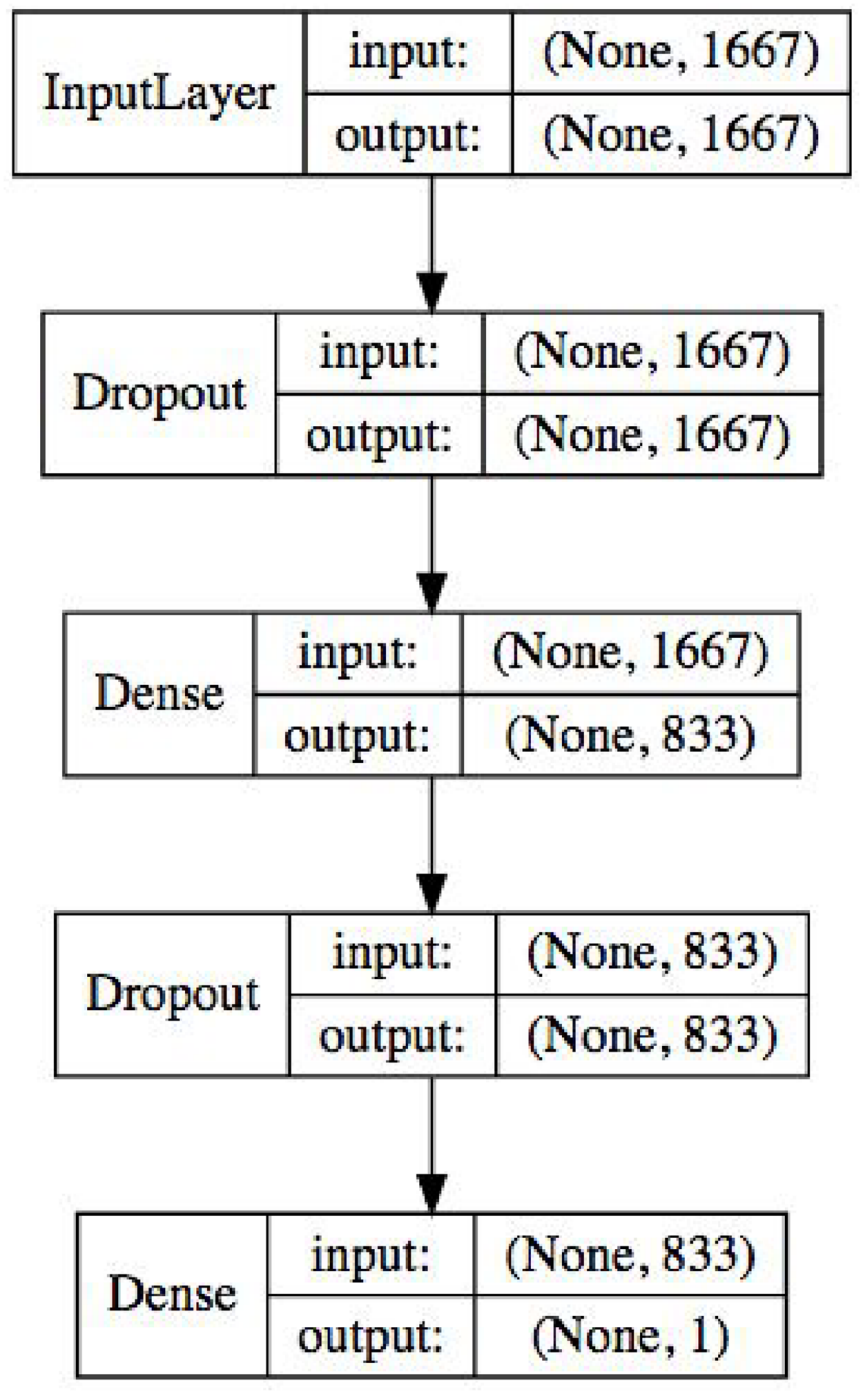
Neural Network model architecture (Note: Layer sizes are assuming all features are used).

After evaluating a variety of neural network architectures, we found the best-performing model to be a two-layer neural network, containing one dense hidden layer with half the size of the input layer, and dropout nodes between all layers to prevent overfitting. Our model architecture can be seen in Fig 3. To train the neural network, we used the keras framework [20] on top of Google’s TensorFlow [21] algorithm. We trained in batches of 64 samples using the Adam optimizer [22], limiting our training to 5 epochs because we found that any further training tended to result in overfitting, as indicated by validation accuracy decreasing with each epoch while training loss continued to improve.

**Fig 4:**
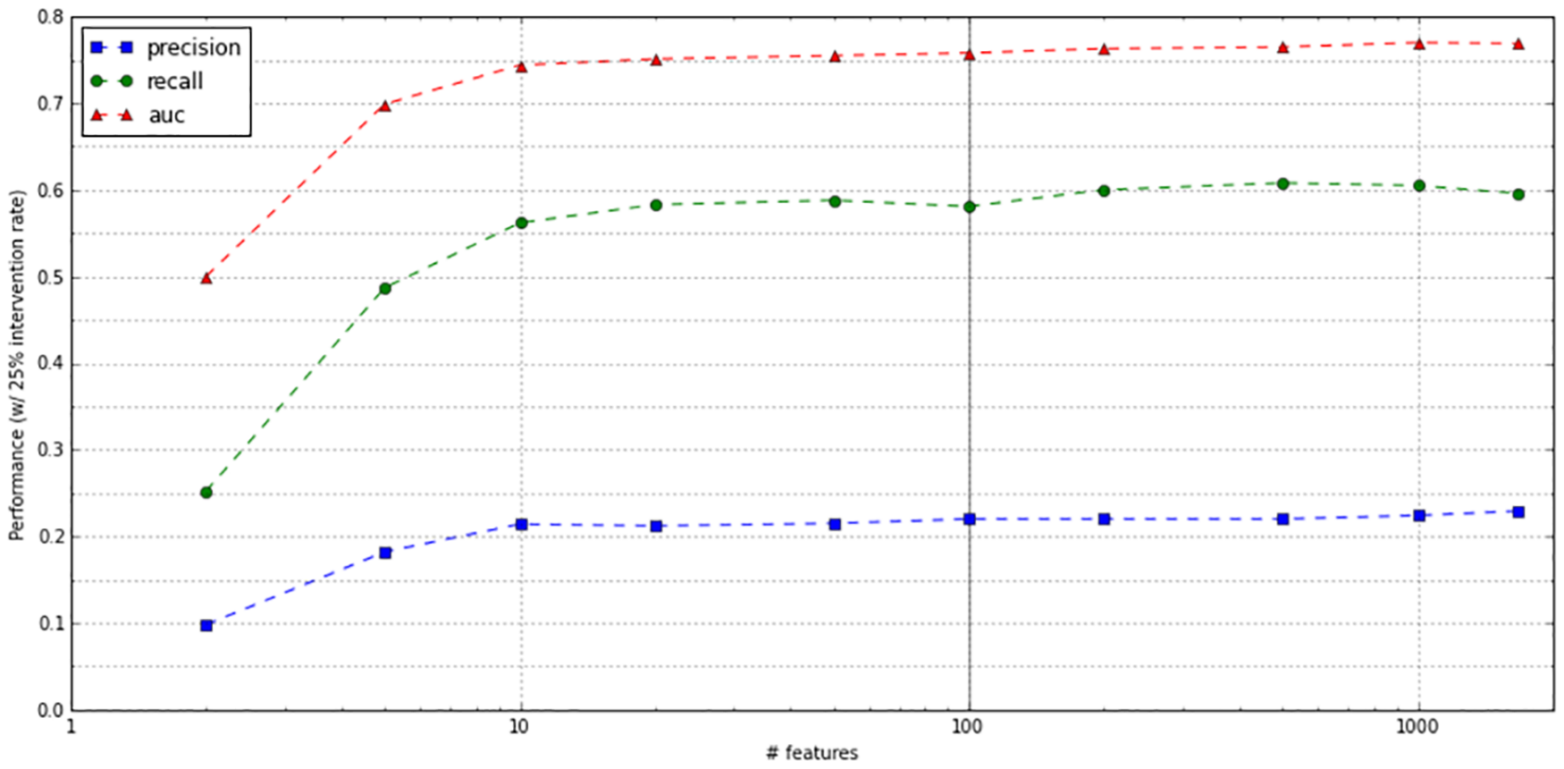
Comparison of NN model performance (with retrospective validation) vs number of features.

Initially, we trained the model on 1667 features extracted from the dataset. We then retrained the model using the top N features most correlated with 30-day readmission, for different values of N. As shown in Fig 4, the model achieved over 95% of the optimal precision when limited to the top 100 features, suggesting that 100 features is a reasonable cutoff for achieving near-optimal performance at a fraction of the training time and model size required for the full model. Table 3 summarizes the features most correlated with readmission risk.

**Table 3:** Top most correlated features with 30-day readmission.

Category: Feature	Linear Correlation
**Utilization: # of inpatient visits in the past 12 months**	0.226
**Utilization: # of inpatient visits in the past 6 months**	0.224
**Comorbidities: Charlson Comorbidity Index (CCI)**	0.215
**Utilization: inpatient visits in the past 3 months**	0.210
**Lab Results: # of lab results marked as ‘low’, ‘high’, or ‘abnormal’**	0.197
**Lab Results: total # of lab results conducted**	0.160
**Lab Results: lab results component of Tabak Mortality Score**	0.157
**Comorbidities: mild liver or renal disease**	0.149
**Utilization: emergency visits component (“E”) of LACE score**	0.143
**Comorbidities: congestive heart failure**	0.143

Measuring a model’s performance cannot be completely separated from its intended use. While one metric, AUC, is designed to measure model behavior across the full range of possible uses, in practice risk models are only ever used to flag a minority patient population, and so the statistic is not fully relevant. Metrics like precision and recall require a yes/no intervention threshold before they can even be computed, something that we lack as this model is not slated for a specific clinical program. For simplification, we assumed the model would be used in an intervention on the 25% of patients with the highest predicted risk. We chose 25% because this is the fraction of patients that LACE naturally flags as high-risk, so we conservatively compare to LACE on its best terms. Additionally, we wanted to understand the predictive power of each set of features. To achieve that, we removed individual feature sets, one at a time, and compared the performance (in terms of AUC) with the best performing model.

Providers often want to focus their interventions on a specific patient population based on their age, geography or medical condition. Therefore, it is important to measure how well the model performs in each of those subpopulations. In addition, so far, CMS has penalized hospitals for excessive readmission of patients with heart failure (HF), chronic obstructive pulmonary disease (COPD), acute myocardial infarction (AMI), or pneumonia^5^. We compared the performance of our model against LACE in each of those subpopulations.

### Cost savings analysis

The main objective of this research study is to build and pilot a predictive model to accurately identify high-risk patients, and support the implementation of valuable and cost-effective post-discharge interventions. Therefore, a cost-saving analysis could assist decision makers to effectively plan and optimize hospital resources.

The optimal intervention threshold for maximizing cost savings depends on (1) the average cost of a readmission, (2) the expected cost of intervention(s), and (3) the expected effectiveness of intervention(s). Then, we can calculate the expected savings from each given intervention strategy as follows:

$$\begin{array}{lll} Savings & = & Expected\;Benefits\;-\;Costs\\ & = & \left(\#\;Correctly-Chosen\;Interventions\right)\times \left(Intervention\;Success\;Rate\right)\\ & & \times \left(Readmission\;Cost\right)-\left(\#\;Total\;Interventions\right)\times \left(Intervention\;Cost\right) \end{array}$$

## Results

Table 4 compares the performance (assuming a 25% intervention rate) of our models and that of LACE when run on all data with 5-fold validation, using the metrics of precision (PPV), recall (sensitivity), and AUC (c-statistic).

**Table 4:** Comparison of the performance of our models with that of LACE, assuming a 25% intervention rate.

Model*	# Features	Precision	Recall	AUC	Training time**	Evaluation time**
*2-layer neural network*	1667	**24%**	60%	**0.78**	2650 sec	154 sec
*2-layer neural network*	500	22%	**61%**	0.77	396	31
*2-layer neural network*	100	22%	58%	0.76	169	14
*Random forest*	100	23%	57%	0.77	669	43
*Logistic regression*	1667	17%	41%	0.66	60	4
*Logistic regression*	100	21%	52%	0.72	17	0.1
*LACE*	4	21%	49%	0.72***	0	0.2

*—Model parameters: neural network (as described in Methods section), random forest (1000 trees of max depth 8, with 30% of features in each tree), logistic regression (default parameters in scikit-learn package)

**—Per-fold training time was measured on a 2014 Macbook Pro with a 4-core 2.2 GHz processor and 16GB RAM. The neural network model ran on four cores, while the other models could only be run on a single core. Training was performed on 259,050 records and evaluation was performed on 64,763 records.

***—We computed the AUC for LACE by comparing the performance of LACE models at every possible threshold. However, LACE is normally used with a fixed threshold, so the given AUC overstates the performance of LACE in practice.

Any model trained on present data will always perform slightly worse on future data, as the world changes and the model’s assumptions become less accurate. To evaluate performance on future data, we trained our best-performing model, the two-layer neural network, on all patients’ data with a hospitalization event prior to 2015, and measured the performance of the model in predicting 30-day readmissions in 2015. As seen in Table 5, a slight performance reduction in precision (from 24% to 23%), relative to the model’s performance on all data, is observed.

**Table 5:** Performance of our model versus LACE on 2015 data when trained on data through 2014.

Model*	# Features	Precision	Recall	AUC	Training time**
*2-layer neural network*	all	**23%**	**59%**	**0.78**	1040 sec
*LACE*	4	19%	50%	0.71***	0

*—Model parameters: neural network (as described in Methods section), random forest (1000 trees of max depth 8, with 30% of features in each tree), logistic regression (default parameters in scikit-learn package)

**—Per-fold training time was measured on a 2014 Macbook Pro with a 4-core 2.2 GHz processor and 16GB RAM. The neural network model ran on four cores, while the other models could only be run on a single core. Training was performed on 259,050 records and evaluation was performed on 64,763 records.

***—We computed the AUC for LACE by comparing the performance of LACE models at every possible threshold. However, LACE is normally used with a fixed threshold, so the given AUC overstates the performance of LACE in practice.

**Fig 5:**
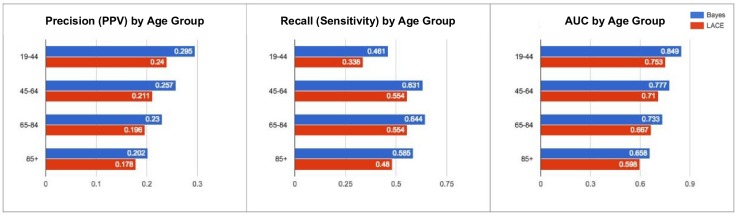
Comparison of artificial neural network model with LACE in 4 different age brackets.

**Fig 6:**
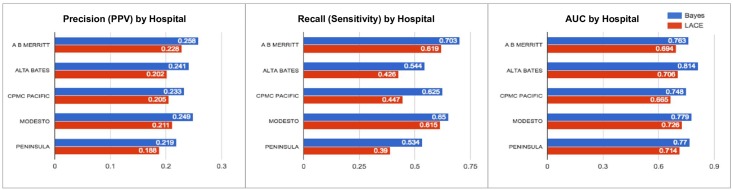
Comparison of the model performance among top five Sutter Health hospitals by the number of inpatient records.

**Fig 7:**
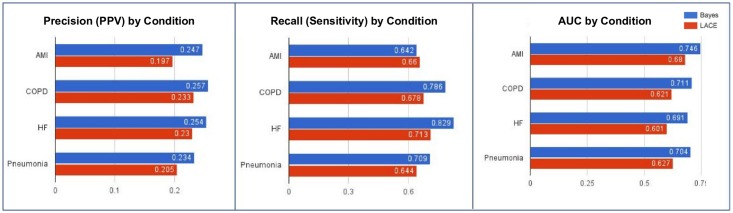
Comparison of the neural network model’s performance among subgroups with varying medical conditions.

Fig 5 compares our model with LACE in four different age brackets. From this graph, the discriminatory power of the model decreases in older patients. However, it still outperforms LACE (+0.02 precision, +0.11 recall). Fig 6 compares the performance of the model in the top five Sutter Health hospitals by number of inpatient records. As seen in this graph, performance varies depending on the hospital location and the population it serves. Lastly, Fig 7 compares our model’s performance among subgroups with varying medical conditions. While the result suggests that the model performs slightly worse in those conditions, it is still superior to LACE (+ 0.03–0.05 precision, + 0.02–0.12 recall).

Due to the nonlinear relationship of different feature sets, it is virtually impossible to calculate the absolute contribution of individual feature sets on the model. However, we can approximate their effect by measuring the model performance using all feature sets except one. The result of this experiment is shown in Table 6. As seen in this table, removing any single feature set, except Medications, Utilization or Vitals, does not have a significant effect on the model performance.

**Table 6:** Comparison of performance of each feature group on the neural network model, tested by withholding one feature group at a time and measuring the impact on model AUC.

Feature Group	Effect on AUC
**Medications**	+ 0.010
**Utilization**	+ 0.007
**Vitals**	+ 0.006
**Lab Results**	+ 0.003
**Discharge**	+ 0.003
**Hospital Problems**	+ 0.002
**Provider**	+ 0.001
**Comorbidities**	+ 0.001
**Basic Demographics**	+ 0.001
**Payer**	+ 0.000
**Health History**	+ 0.000
**Admission**	+ 0.000
**Encounter Reason**	– 0.001
**Socioeconomic**	– 0.002
**Procedures**	– 0.005

For the cost savings analysis, while the actual values may be difficult (or, in some cases, even impossible) to predict, we will use the following values as an example: Readmission cost: $5000, Intervention Cost: $250, Intervention success rate: 20%.

**Fig 8:**
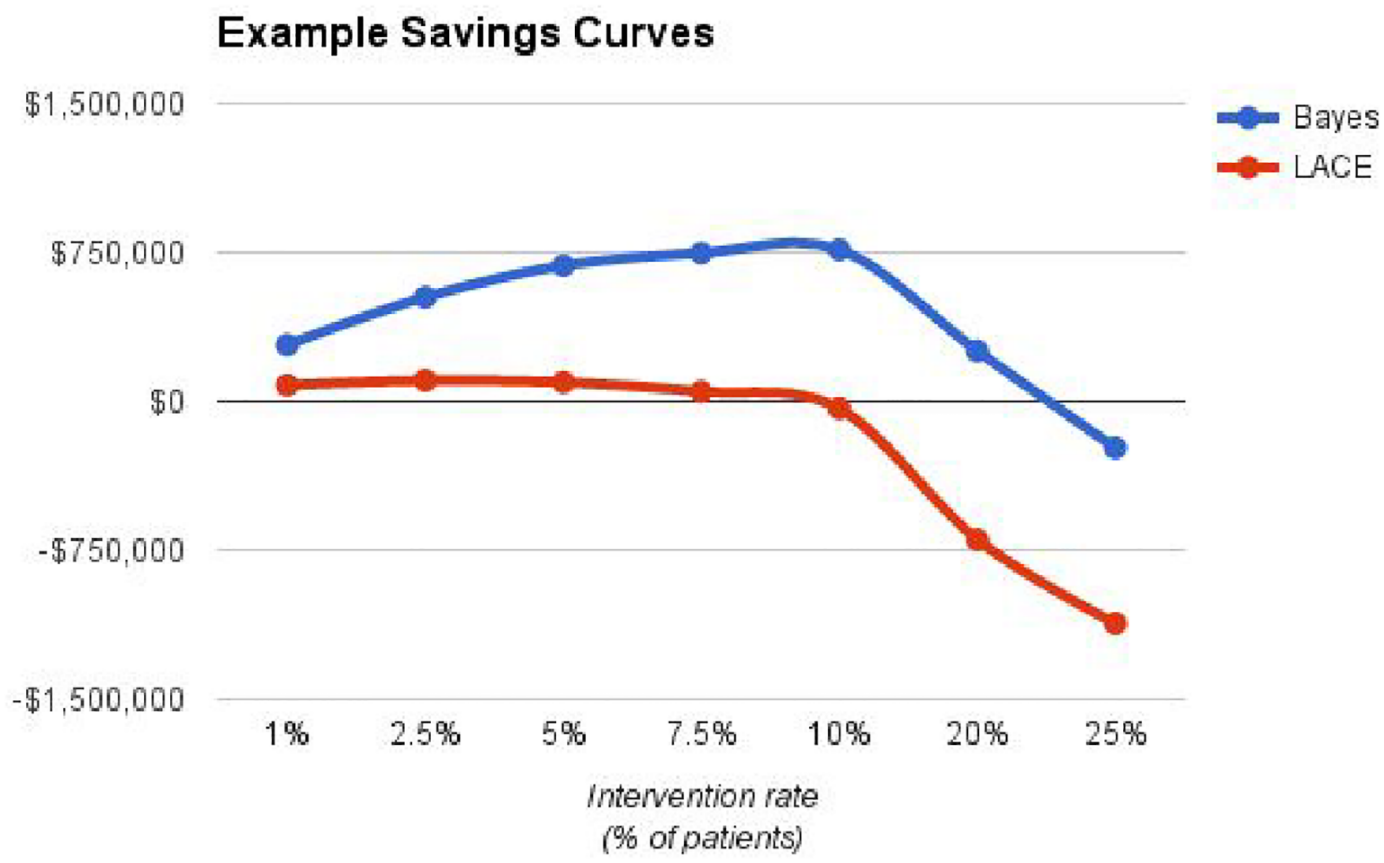
The projected saving values as a function of the intervention rate, with the example parameters given for the cost-savings analysis in the results section.

Fig 8 shows the projected saving values as a function of the intervention rate (percentage of patients subjected to readmission-prevention interventions).

## Discussion

The factors behind hospital readmission are numerous, complex and interdependent. Although some factors, such as prior utilization, comorbidities, and age, are very predictive by themselves, improving the predictive power beyond LACE requires models that capture the interdependencies and non-linearity of those factors more efficiently. Artificial neural networks (ANN), by modeling nonlinear interactions between factors, provide an opportunity to capture those complexities. This nonlinear nature of ANNs enables us to harness more predicitive power from the additional extracted EHR data fields beyond LACE’s four parameters.

Furthermore, neural networks are compact and can be incrementally retrained on new data to avoid the “model drift” that occurs when a model trained on data too far back in the past performs progressively worse on future data that follows a different pattern.

The TensorFlow framework provides several added benefits for training a readmission model. First, TensorFlow can run in a variety of environments, whether on CPUs, GPUs, or distributed clusters. This means that the same kind of model can be trained in a variety of different hospital IT architectures, and achieve optimal performance in each. Secondly, with the aid of high-level interfaces, such as keras, TensorFlow can model neural network architectures in a very natural way. This enabled us to quickly experiment with different neural network setups to find the ideal configuration for the problem. Finally, TensorFlow is an actively maintained open-source project, and its performance improves continually through contributions from the open-source machine-learning community.

A fair comparison of our model with results in existing literature is not feasible, because the performance of readmission risk models varies tremendously between different patient populations, and no previous readmission prediction work has been done on the Sutter Health patient population. Even the LACE model’s performance varies in the literature from 0.596 AUC [10] to 0.684 AUC [11], which illustrates the impact of patient population on the accuracy of readmission prediction.

The performance of our model (as measured by precision, recall, and AUC) within patient subgroups tends to be worse than the performance of the same model within the whole patient population. Some of this performance drop can be explained by the fact that each subgroup represents a reduced feature set to our model—for example, age is no longer as predictive a feature to when every patient in a subgroup has a similar age. Furthermore, our model tends to perform on subgroups that LACE also has the worst performance on, such as patients aged 85+ (Fig 5) or patients with heart failure (Fig 7), suggesting that certain patient subpopulations have significantly less predictable readmission patterns than the general patient population.

We used two sources of SDoH features: health history questions (regarding tobacco, alcohol, and drug use) and block-level census data based on patient address. The health history features had some predictive value, two of them (“no alcohol use” and “quit smoking”) being in the top 100 features most linearly correlated with readmission risk. However, the census features were less predictive, with no features in the top 100 and only a few in the top 200 (such as poverty rate and household income). Both feature sources suffered from drawbacks: the health surveys were both brief and incomplete for ~25% of patients, while the block-level census data only provided information about a patient’s neighborhood but not about the patient themselves. For SDoH features to provide significant predictive value, they would have to be both comprehensive and individualized.

Since this study was conducted on EHR data from Sutter Health network of hospitals in California, it does not capture potential out-of-network hospital readmissions. To address this limitation, the dataset could be supplemented by state or national index hospital admissions to build a more comprehensive dataset.

## Conclusions

In this study, we successfully trained and tested a neural network model to predict the risk of patients’ rehospitalization within 30 days of their discharge. This model has several advantages over LACE, the current industry standard, and other proposed models in the literature including (1) significantly better performance in predicting the readmission risk, (2) being based on real-time data from EHR, and thus applicable at the time discharge from hospital, and (3) being compact and immune to model drift. Furthermore, to determine the classifier’s labeling threshold, we suggested a simple cost-saving optimization analysis.

Further research is required to study the effect of more granular and structured social determinants of health data on the model’s predictive power. Some studies [23] have shown that natural language processing (NLP) techniques could be used to extract SDoH data from patient’s case notes. However, the most systematic method is to gather such data from SDoH screeners. Currently, multiple initiatives [24] are underway to standardize SDoH screeners, and integrate them into EHR systems.

The importance of reducing hospital readmissions, and therefore risk assessment, is likely to only grow in importance in the years to come. We believe that predictive analytics in general, and modern machine-learning techniques in particular, are powerful tools that have to be fully exploited in this field.

## Software release

The neural network model described in the paper, as well as the code to run it on EMR data, is available (under the Apache license) at https://github.com/bayesimpact/readmission-risk.
