# Correction: Predicting all-cause risk of 30-day hospital readmission using artificial neural networks

**DOI:** 10.1371/journal.pone.0197793

**Published:** 2018-05-17

**Authors:** Mehdi Jamei, Aleksandr Nisnevich, Everett Wetchler, Sylvia Sudat, Eric Liu, Kirtan Upadhyaya

Dr. Kirtan Upadhyaya is not included in the author byline. He should be listed as the sixth author, and his affiliation is 1: Bayes Impact, Technology 501(c)(3) Non-profit, San Francisco, California, United States of America. The contributions of this author are as follows: Conceptualization, Investigation, Validation, and Writing – Review & Editing.

The last five authors, Aleksandr Nisnevich, Everett Wetchler, Sylvia Sudat, Eric Liu, and Kirtan Upadhyaya, should be noted as contributing equally to this work.

The correct citation is: Jamei M, Nisnevich A, Wetchler E, Sudat S, Liu E, Upadhyaya K (2017) Predicting all-cause risk of 30-day hospital readmission using artificial neural networks. PLoS ONE 12(7): e0181173. https://doi.org/10.1371/journal.pone.0181173.
